# Bioluminescence microscopy using a short focal-length imaging lens

**DOI:** 10.1111/jmi.12109

**Published:** 2014-01-03

**Authors:** K Ogoh, R Akiyoshi, May-Maw-Thet, T Sugiyama, S Dosaka, Y Hatta-Ohashi, H Suzuki

**Affiliations:** * Corporate Research and Development Center, Olympus CorporationKuboyama, Hachioji, Tokyo, Japan; † Department of Anesthesiology, Faculty of Medicine, Kagawa UniversityIkenobe, Miki-cho, Kita-gun, Kagawa, Japan

**Keywords:** Bioluminescence microscopy, imaging lens, short focal-length

## Abstract

Bioluminescence from cells is so dim that bioluminescence microscopy is performed using an ultra low-light imaging camera. Although the image sensor of such cameras has been greatly improved over time, such improvements have not been made commercially available for microscopes until now. Here, we customized the optical system of a microscope for bioluminescence imaging. As a result, bioluminescence images of cells could be captured with a conventional objective lens and colour imaging camera. As bioluminescence microscopy requires no excitation light, it lacks the photo-toxicity associated with fluorescence imaging and permits the long-term, nonlethal observation of living cells. Thus, bioluminescence microscopy would be a powerful tool in cellular biology that complements fluorescence microscopy.

## Introduction

Bioluminescence assays based on the firefly luciferin–luciferase reaction have been widely developed in the fields of analytical and clinical chemistry as a bioluminescent reporter of chemical reactions associated with ATP metabolism (DeLuca, 1978; DeLuca & McElroy, 1986; LaRossa, 1998). Since the cloning of the firefly luciferase gene, luciferase has been used as a reporter enzyme to assay the activity of a particular gene promoter (de Wet *et al.* 1987; Brasier *et al.* 1989; Alam & Cook, 1990) because it offers greater sensitivity and operational simplicity compared with chloramphenicol acetyl transferase or beta galactosidase assays; moreover, the luciferase system does not require use of radioactive isotopes. In the luciferase assay, the intensity of emitted light from cells or cell lysates is measured with a luminometer. Therefore, it is impossible to simultaneously monitor promoter activity and cellular characteristics in the same cell as image with this luminometer method.

Time-lapse image analysis of promoter activity (gene expression) and cellular characteristics in single live cells is essential for the study of cell proliferation and differentiation, especially microscopic studies of morphogenesis. For such live-cell imaging studies, long-term observation is required in healthy cells and tissues. In addition, light excitation and associated phototoxicity in fluorescence assays are not a factor in bioluminescence reactions, thereby precluding background autofluorescence and toxicity. Thus, bioluminescence imaging is ideal for long-term observations of single live cells.

Bioluminescence image analysis of promoter activity at the single-cell level has been performed using microscopes equipped with ultra low–light imaging cameras, such as liquid nitrogen–cooled charge-coupled device (CCD) cameras, photon-counting CCD cameras and image-intensifying CCD cameras (Frawley *et al.* 1994; Thompson *et al.* 1995; White *et al.* 1995; Castaño *et al.*, 1996; Kennedy *et al.* 1997; Takasuka *et al.* 1998; Maire *et al.* 2000; Welsh *et al.* 2004; Masamizu *et al.* 2006). However, image acquisition time is too lengthy for the observation of cellular biological events, or image resolution is too low for detection of single cells compared with that of conventional CCD cameras. Therefore, the satisfactory analysis of bioluminescence images at the single-cell level has not been attained. Recently, electron multiplying CCD (EM-CCD) cameras, which yield higher sensitivity and image quality than previous ultra low–light imaging cameras, were commercially released and used for bioluminescence microscopy (Hoshino *et al.* 2007; Kwon *et al.* 2010; Suzuki *et al.* 2011). Although the image sensor of ultra low–light imaging cameras has been greatly improved over time, such improvements have not been made commercially available for microscopes until now.

Bioluminescence imaging is based on the detection of light emitted by living cells expressing a luciferase gene or other luminescence-related gene. Conventional fluorescence microscopes are inefficient at transmitting light from the sample to the detector, necessitating long exposure times. Generally, the degree of brightness (*I*) of an image is directly proportional to the square of the numerical aperture (*NA*) of the objective lens and inversely proportional to the square of magnification (*M*) of the image. This can be represented as *I*∝(*NA*/*M*)^2^. Therefore, a higher *NA* and lower *M* yield much brighter images; however, it is difficult to obtain both conditions. The higher the *NA* of the objective lens, the shorter the working distance (shorter focal length) and *M* of the image is calculated by the focal-length ratio of the imaging lens (*F*_i_) to objective lens (*F*_o_) in an infinity optical system. This can be represented as *M* = *F*_i_/*F*_o_. Thus, high *NA* and low *M* are mutual trade-offs. On the other hand, the value of *NA*/*M* is the same as *NA* of the imaging lens (tube lens), geometrically as denoted *NA*′. Therefore, a microscope with a high *NA*′ (short focal-length imaging lens) makes it possible to achieve a higher *NA* and lower *M* without further improvement of the objective lens. Thus, we demonstrated that higher value of *I* (*I* > 0.01) is required for bioluminescence microscopy of single live cells (Suzuki *et al.* 2007).

In this study, we customized the short focal-length of an imaging lens for bioluminescence microscopy and performed bioluminescence imaging of live cells expressing the beetle luciferase gene using a conventional colour and EM CCD cameras. Furthermore we used the deep-sea shrimp luciferase, which is 150-fold brighter than beetle luciferase (Hall *et al.* 2012), for organelle-targeted imaging to show spatial resolution of this system. Throughout this study, we were faced with unique characteristic of bioluminescence differed from fluorescence for imaging. Therefore, spectral properties of luciferase expressed in live cells were also presented.

## Materials and methods

### Bioluminescence microscope

**Fig 1:**
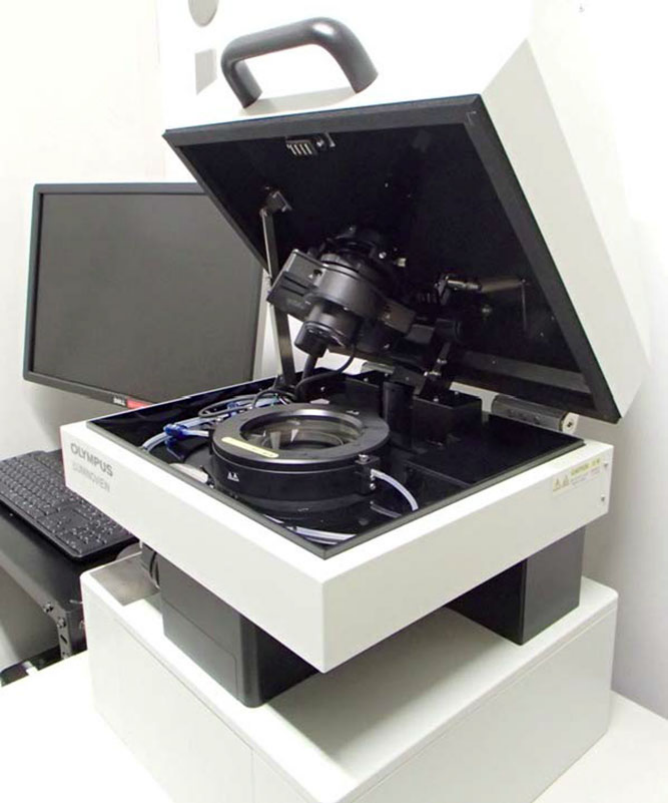
Bioluminescence microscope, LV200. The stage-top incubator with temperature and CO_2_ gas controller was added on the sample stage. The observation area was covered with a dark box.

Figure 1 shows the inverted bioluminescence microscope used in our studies (Luminoview LV200; Olympus, Tokyo, Japan). Bioluminescence emitted from live cells in a culture dish was collected by an objective lens and the light passed through an imaging lens that then transmitted the image to a CCD camera. The objective lenses used in this study were UPlanFLN 40×/NA 1.30 Oil and UPlanFLN 100×/NA 1.30 Oil (Olympus). The DP70 colour CCD camera (Olympus) and ImagEM EM-CCD camera (C9100–13; Hamamatsu Photonics, Shizuoka, Japan) were equipped for an LV200 microscope. The imaging lens was developed with a focal length of 36 mm with an *NA* of 0.2. A stage-top incubator with temperature and CO_2_ gas controllers (MI-IBC-IF; Tokai Hit Co., Shizuoka, Japan) was added to the sample stage. The observation area was covered with a dark box.

### Bioluminescence imaging

Luciferase genes originating from the firefly (Luc2), click beetle (CBG99 and CBR) or deep-sea shrimp (NanoLuc; Promega, Madison, WI, USA) were inserted into the pcDNA 3.1 mammalian expression vector (Invitrogen, Carlsbad, CA, USA). The luciferase expression vector construct was transfected into U2OS human osteosarcoma cells (ATCC, Manassas, VA, USA) using the FuGene HD transfection reagent (Roche, Basel, Switzerland). A stable cell line expressing each luciferase gene was established in Dulbecco's Modified Eagle's Medium (DMEM; Invitrogen) containing 10% foetal bovine serum (FBS) and 1 mg mL^–1^ geneticin for selection of stably transfected cells.

U2OS cells stably expressing the luciferase gene were cultured on 35 mm glass-bottomed dishes in DMEM containing 10% FBS. Then, the three beetle luciferase cell lines (CBG99, CBR, Luc2; 1.0 × 10^5^ cells dish^–1^) and NanoLuc cell line (3.0 × 10^5^ cells dish^–1^) were cultured overnight. Beetle d-luciferin potassium salt (Promega; 1 mM, for beetle luciferase) or furimazine (Promega; 12.5 μM for deep-sea shrimp luciferase) was added. Bioluminescence imaging of cells was performed on cells kept at 25°C or 37°C with 5% CO_2_ using an LV200 microscope equipped with a UPlanFLN 40× Oil objective lens and DP70 colour CCD camera. Binning of the CCD was 1 × 1 (1360 × 1024 pixels), International Organization for Standardization gain was 1600, and the exposure time for beetle cells was 2 min and for NanoLuc cells was 10 s, with 3 and 2 min intervals, respectively, over the course of 3 h. A bioluminescence image was generated after subtraction of a blank image of the same exposure time. Then, the luminescence intensity in each cell line was measured using image acquisition and analysis software (AquaCosmos; Hamamatsu Photonics).

To show usefulness of LV200 for acquiring a brighter bioluminescence image, a conventional inverted microscope (IX70, Olympus) was used for comparison of bioluminescence image with the same conditions (stable cell lines, objective lens, CCD camera and exposure time).

### Spectral analysis

The four cell lines (3.0 × 10^5^ cells plate^–1^) were suspended in DMEM containing 10% FBS and incubated in a CO_2_ (5%) incubator at 25°C or 37°C. After addition of beetle luciferin (1 mM) or furimazine (12.5 *μ*M), bioluminescence spectra were immediately obtained with a luminescence spectrometer (AB-1850; ATTO, Tokyo, Japan). This instrument uses a diffraction grating system to spectrally decompose light, which then transmits the image to a high-sensitivity CCD camera. The slit width was 0.5 mm, and the exposure times were 5 and 1 min for beetle and NanoLuc cell lines, respectively. Bioluminescence intensity between 25 and 37°C, and among cell lines at 37°C, was compared with the total surface area that emitted luminescence in the relevant spectrum for luciferin or furimazine.

### Organelle targeting

NanoLuc was used as a tag for organelle localization, similar to a fluorescent protein. Enhanced yellow and cyan fluorescent protein (EYFP and ECFP) genes of the pEYFP-Nuc and pECFP-Mito vectors (GE Healthcare, Buckinghamshire, UK), which contains a nuclear localization sequence (NLS) at the 3′ end of Enhanced yellow fluorescent protein gene and a mitochondrial targeting sequence (subunit VIII of human cytochrome C oxidase, CoxVIII) at the 5′ end of Enhanced cyan fluorescent protein gene, respectively, were replaced with the NanoLuc gene. Furthermore, the CoxVIII was copied tandem three repeats. For endoplasmic reticulum targeting, NanoLuc gene containing a sequence of calreticuin at the 5′ end and an endoplasmic reticulum retrieval sequence (KDEL) at the 3′ end was inserted into pcDNA 3.1. Then, U2OS cells were transiently transfected with these constructs using FuGene HD. Before substrate addition (12.5 *μ*M furimazine) cells were washed with culture medium three times, and images of the organelle labelled with NanoLuc were captured by LV200 with ImagEM EM-CCD camera and UPlanFLN 100× Oil objective lens at 37°C.

## Results

### Bioluminescence image and spectrum

**Fig 2:**
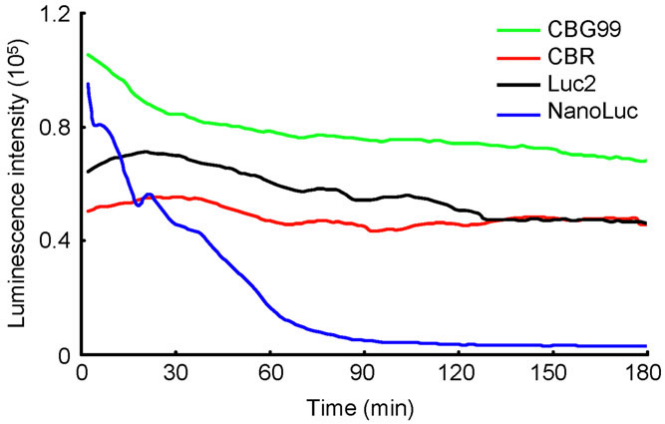
Time course of bioluminescence intensity from U2OS cells of four lines expressing luciferase CBG99, CBR, Luc2 or NanoLuc. Cells were incubated in DMEM containing 10% FBS with 5% CO_2_ at 37°C. The substrate was 1 mM beetle luciferin for beetle luciferase or 12.5 *μ*M furimazine for NanoLuc.

**Fig 3:**
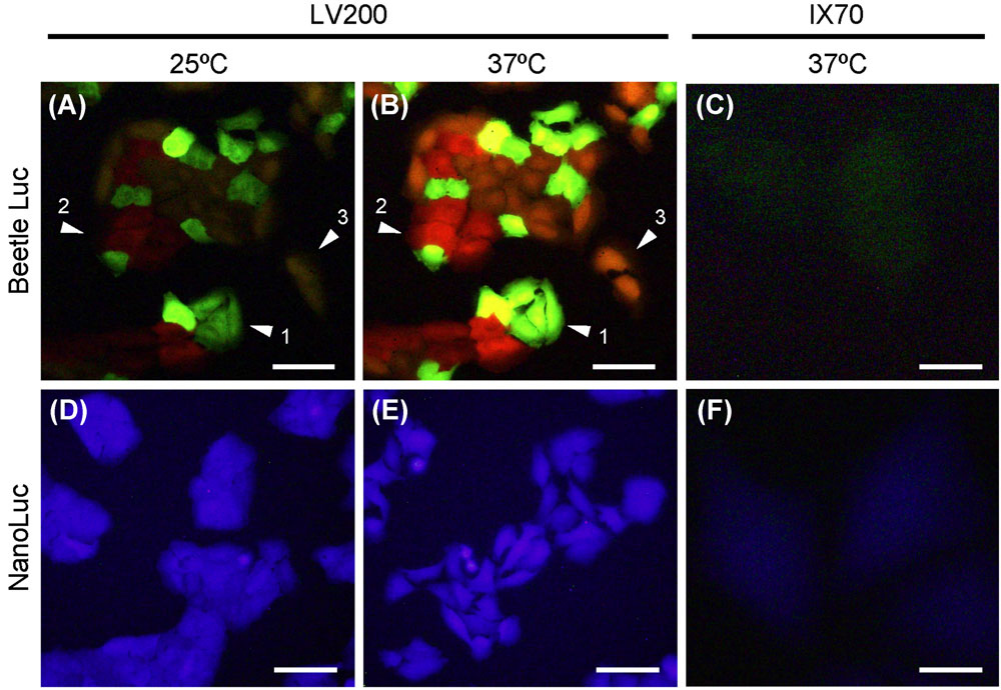
Bioluminescence images of U2OS cell lines expressing beetle luciferases (CBG99, CBR, Luc2) or NanoLuc at 25 and 37°C. Cells were incubated in DMEM containing 10% FBS with 5% CO_2_. The substrate was 1 mM beetle luciferin for beetle luciferase or 12.5 *μ*M furimazine for NanoLuc. Images (A, B, D, E) were captured using an LV200 microscope with UPlanFLN 40× Oil objective lens (*NA* = 1.30, *I* = 0.026) and DP70 colour CCD camera. Exposure time was 2 min for beetle luciferase or 10 sec for NanoLuc cell line. The arrowheads denote 1, 2 and 3 cells expressing CBG99, CBR and Luc2 luciferase, respectively. Images (C, F) were captured using an IX70 microscope with UPlanFLN 40× Oil objective lens (*NA* = 1.30, *I* = 0.001) and DP70 colour CCD camera. Exposure time was 10 min for beetle luciferase or 1 min for NanoLuc cell line. All the images were displayed with no level adjustment. Scale bars, 100 *μ*m (A, B, D, E) and 20*μ*m (C, D).

Figure 2 shows the time course of bioluminescence intensity from U2OS cells arbitrarily selected from four cell lines expressing CBG99, CBR, Luc2 and NanoLuc luciferase at 37°C based on time-lapse images. The intensity was relatively stable in the beetle luciferase cell lines (CBG99, CBR, Luc2) for 3 h but immediately decreased after addition of substrate in the NanoLuc cell line. Therefore, images were captured at 1 h and at 10 min after substrate addition to the beetle and NanoLuc cell lines, respectively (Figure 3).

**Fig 4:**
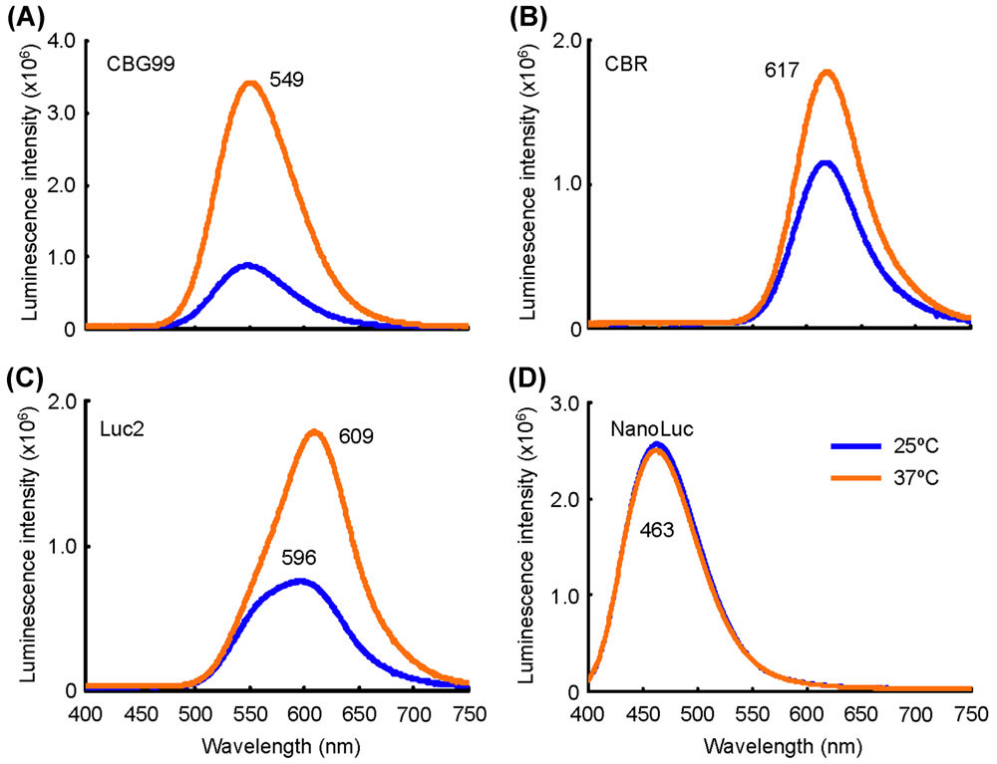
Bioluminescence spectra of U2OS cell lines expressing luciferase CBG99 (A), CBR (B), Luc2 (C) or NanoLuc (D) at 25 and 37°C. Cells were incubated in DMEM containing 10% FBS with 5% CO_2_. The substrate was 1 mM beetle luciferin for beetle luciferase or 12.5 *μ*M furimazine for NanoLuc.

Figures 3(A) and 3(B) show three cell lines expressing CBG99, CBR and Luc2 luciferase at 25 and 37°C. These images were clearly captured within 2 min using a conventional DP70 colour CCD camera. CBG99 and CBR cell lines showed no colour shift with temperature at maximal wavelengths of 549 and 617 nm, respectively; however, luminescence intensity increased in CBG99 and CBR cells by 3.7- and 1.5-fold, respectively, with increasing temperature (Fig.4A, B, Table 1). On the other hand, the Luc2 cell line showed a colour shift from yellow (596 nm) at 25°C to orange (609 nm) at 37°C, and luminescence intensity also increased 2.0-fold with increasing temperature (Figs. 3A, B and 4C, Table 1). Figures 3(D) and (E) show one cell line expressing NanoLuc luciferase at 25 and 37°C. The images were captured within 10 s; thus, the luminescence intensity of the NanoLuc cell line was much brighter than that of the beetle luciferase cell lines although they required different substrates. The NanoLuc cell line showed no shift in colour (463 nm) or intensity with increasing temperature (Fig. 4D, Table 1). Relative luminescence intensities among the beetle luciferase cell lines (under the same conditions of cell number and substrate) were summarized in Table 1.

**Table 1:** Spectral properties of beetle (CBG99, CBR, Luc2) and deep-sea shrimp (NanoLuc) luciferases expressed in U2OS cell lines

	λ Max (nm)			
			Relative Intensity	Relative Intensity/Luc2
Cell Line	25°C	37°C	(37°C/25°C)	(37°C)
CBG99	548.69 (±0.95)	549.93 (±0.36)	3.71 (±0.24)	1.72 (±0.11)
CBR	616.62 (±0.00)	617.44 (±0.94)	1.51 (±0.45)	0.83 (±0.25)
Luc2	596.47 (±0.36)	608.80 (±0.71)	2.02 (±0.87)	1.00 (±0.43)
NanoLuc	463.22 (±0.63)	462.59 (±1.09)	0.98 (±0.25)	-

*Note*: Bioluminescence intensity between 25 and 37°C, and among cell lines at 37°C, was compared with the total surface area that emitted luminescence in the relevant spectrum. Mean ± SD, *n* = 3.

Next, we used a conventional inverted microscope (IX70) to compare the bioluminescence image with that of LV200 using the stable cell lines established. As a result, bioluminescence image could not be captured with the same conditions (stable cell lines, objective lens, CCD camera and exposure time), but it took 10 and 1 min exposure time (5- to 6-fold longer exposure time) to raise an image for beetle luciferase and LanoLuc cell lines, respectively (Fig. 3C, F). Although a blank image subtraction was performed, 10 min was upper limit of expose time for DP70 colour CCD camera due to intense background elevation. All the images in Figure 3 were displayed with no level adjustment for comparison.

### Organelle targeting

**Fig 5:**
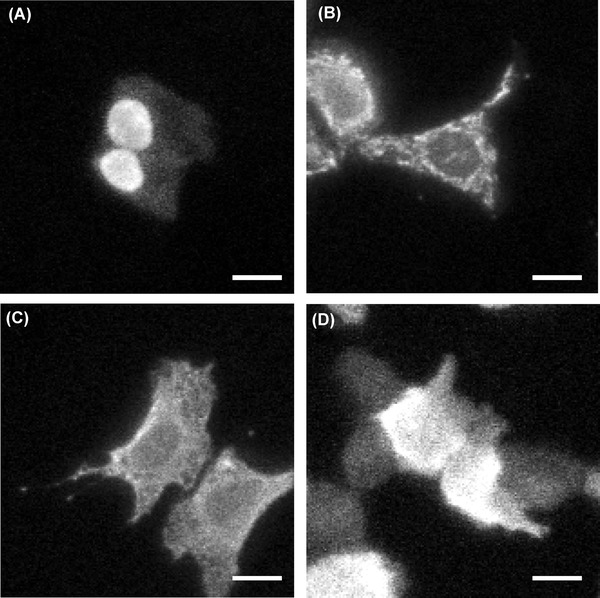
Bioluminescence images of NanoLuc fused with NLS (A), CoxVIII (B), calreticulin (C) or no targeting sequence (D) in U2OS cells. Images were captured using an LV200 microscope with UPlanFLN 100× Oil objective lens and ImagEM EM-CCD camera at 37°C. Exposure time, 300 ms (A, D), 500 ms (B) and 1 sec (C); Furimazine, 12.5 *μ*M; Scale bars, 20 *μ*m.

To show spatial resolution of the bioluminescence image of LV200, organelle targeted images were captured. Figure 5 shows bioluminescence images of NanoLuc fused with NLS (A), CoxVIII (B), calreticulin (C) or no targeting sequence (D) in U2OS cells with 300 ms to 1 sec exposure time. The NanoLuc-NLS accumulated in the nucleus of the cell, and the CoxVIII-NanoLuc and calreticulin-NanoLuc-KDEL appeared in a meshwork pattern in the cytoplasm. Thus, the nucleus and cytoplasm were discriminated clearly, and mitochondria and endoplasmic reticulum was recognized in the cytoplasm.

## Discussion

As shown in Figure 3, bioluminescence images of cells expressing the luciferase gene at 37°C can be clearly captured using an LV200 microscope with 40× objective lens and colour CCD camera. In this case, *M* of the image is reduced by a power of 8 owing to the short focal-length imaging lens, and the value of *I* of this system is 0.026. However, a bioluminescence image cannot be captured by an IX70 using the same objective lens (*I* = 0.001) within the same exposure time. To equalize *I* value between the microscopes, a low *M* and high *NA* objective lens (e.g. 8×, *NA* 1.30) is required for IX70. However, such high *NA* objective lens is not purchasable commercially. Generally as an imaging lens (e.g. *F*_i_ = 180 mm, Olympus) is fixed in the body of the microscope, it is difficult to achieve low *M* and high *NA*. Thus, a short focal-length imaging lens makes bioluminescence microscopy possible with the use of a conventional objective lens and colour CCD camera.

Figure 5 shows bioluminescence images of NanoLuc fused with organelle. The organelle targeting images are not clearer than fluorescence images using fluorescent proteins previously reported (Rizzo *et al*., 2010). Because total *M* is reduced to 0.2-fold in LV200 system. However, nuclear and cytoplasm are clearly discriminated, and the spatial resolution of LV200 is enough for imaging promoter assay of single live cells (Suzuki *et al.* 2007). Furthermore, use of LV200 with EM-CCD camera and NanoLuc allows the exposure time for bioluminescence imaging of cells and organelle to be reduced by as much as 300 ms to 1 sec, and this system permits luciferase to be used for the detection of protein localization, similar to fluorescent proteins (Hall *et al*., 2012).

Figure 3 shows that an LV200 permits multicolour imaging of luciferses. However, bioluminescence spectra of Luc2 show shifts to longer wavelengths and increases in brightness with increasing temperature from 25 to 37°C (Figs. 3, 4; Table 1; Zhao *et al.* 2005). Enzyme activity–induced long-wavelength shifts, but reductions in quantum yield with decreasing pH or increasing temperature, have been demonstrated by *in vitro* bioluminescence reactions using purified luciferin and luciferase (Seliger & McElroy, 1960, 1964). Furthermore the optimum temperature of firefly luciferase luminescence is 23–25°C (McElroy & Strehler, 1949). According to the Promega Technical Manual for Luciferase Reporter Vectors, Luc2 and other luciferase genes are codon-optimized for gene expression in mammalian cells. Therefore, it has been speculated that luminescence intensity at 37°C is greater that at 25°C although shifts to longer wavelengths occur with reductions in quantum yield. Generally, shifts to longer wavelengths in firefly bioluminescence spectra, but not in the crick beetle, occur with decreasing pH *in vitro* (Viviani & Bechara, 1995). Therefore, spectra changes in firefly luciferase, which is originally cloned and expressed in live cells, also occur with increasing temperature, similar to Luc2 cell lines (Figs. 3A, B and 4C). When using firefly luciferase for multicolour imaging assays, temperature is important for choosing the appropriate optical filter that is required to separate different luminescent signals. This is one way that bioluminescence differs from fluorescence imaging.

## Conclusion

Our current study presents the concept of bioluminescence microscopy using a short focal-length imaging lens, and this system allows the capture of bioluminescence images of cells and organelles by using luciferase, which is comparable with fluorescence microscopy using a fluorescent protein. Because bioluminescence microscopy requires no excitation light, it lacks the phototoxicity and background autofluorescence problems associated with fluorescence imaging and permits the long-term, nonlethal observation of living cells. Thus, bioluminescence microscopy is a powerful tool in cellular biology that complements fluorescence microscopy.
